# Advancements and challenges of artificial intelligence in dermatology: a review of applications and perspectives in China

**DOI:** 10.3389/fdgth.2025.1544520

**Published:** 2025-08-13

**Authors:** Jiaao Yu, Io Hong Cheong, Zisis Kozlakidis, Hui Wang

**Affiliations:** ^1^ School of Medicine, Shanghai Jiao Tong University, Shanghai, China; ^2^ School of Public Health, Shanghai Jiao Tong University, Shanghai, China; ^3^ International Agency for Research on Cancer, World Health Organization, Lyon, France

**Keywords:** artificial intelligence, dermatology, China, AI-aided diagnosis, machine learning

## Abstract

The diagnosis of skin diseases can be challenging due to their diverse manifestations, while early detection of malignant skin cancers greatly improves the prognosis, highlighting the pressing need for efficient screening methods. In recent years, advancements in AI have paved the way for AI-aided diagnosis of skin lesions. Furthermore, the COVID-19 pandemic has spurred the demand of telemedicine, accelerating the integration of AI into medical domains, particularly in China. This article aims to provide an overview of the progress of AI-aided diagnosis in Chinese dermatology. Given the widespread use of public datasets in the reviewed studies, we compared the performance of AI models in segmentation and classification on public datasets. Despite the promising results of AI in experimental settings, we recognize the limitations of these public datasets in representing clinical scenarios in China. To address this gap, we reviewed the studies that used clinical datasets and conducted comparative analyses between AI and dermatologists. Although AI demonstrated comparable results to human experts, AI still cannot replace dermatologists due to limitations in generalizability and interpretability. We attempt to provide insights into improving the performance of AI through advancements in dataset quality, image pre-processing techniques, and integration of medical data. Finally, the role that AI will play in the medical practice and the relationship between AI and dermatologists are discussed. This systematic review addresses the gap in evaluating AI applications in Chinese dermatology, with a focus on dermatological datasets and real-world application.

## Introduction

Dermatologists rely heavily on visual features of skin lesions in their diagnostic process, which can sometimes lead to errors, especially for inexperienced clinicians (1). Accurate dermatological diagnosis builds on years of clinical practice. Studies comparing the performance of different levels of Chinese dermatologists showed that the accuracy of dermatologists with high levels of expertise was between 85% to 95%, while the accuracy of dermatology residents ranged from 60% to 70% (2, 3). Because of the rarity of some unique cutaneous diseases and the similarity between different skin lesions, the misdiagnosis happened even in the tertiary care centers where expertise is anticipated to accrue quicker due to the high volume of cases presented (4–6). What's more, skin diseases were the seventh causes of global disease burden in 2019, especially in the middle Sustainable Development Index (SDI) level countries such as China (7, 8). According to the Global Burden of Disease (GBD), skin and subcutaneous diseases ranked fourth leading cause of disability burden worldwide (9). Analysis based on GBD showed that the burden of disability in China had been growing continuously in the period of 1990 to 2019, with the prevalence of skin disease in the general population increasing by 5.5% in the same period, affecting many millions (8). Therefore, an objective, standardized and efficient assistant tool for dermatologic diagnosis is in need.

Artificial Intelligence (AI) refers to a machine learning-based system that simulates human cognition and can perform tasks typically requiring human intelligence. These tasks include learning from datasets, recognizing patterns, making decisions, understanding natural language, and solving complex problems (10). The application of AI in medical fields is rapidly growing, including but not limited to prediction, diagnosis, treatment, and long-term healthcare (11). Among all, the image diagnosis of AI seemed to be one of the most promising applications, attracting a growing interest from computer scientists and healthcare providers (12). In recent years, an increasing number of studies revealed the potential of AI in promoting the accuracy of skin lesion classification, ranging from malignant skin cancers to inflammatory skin diseases (13). However, although the future looks promising, the use of AI in dermatological diagnosis still faces challenges in everyday clinical practice, including dataset biases, poor interpretability, and ethical issues (14, 15).

Telemedicine, as defined by the World Health Organization (WHO), involves the delivery of health-care services over distance (16). While it cannot fully replace conventional medical care, telemedicine has been proved to be capable of improving medical services in terms of diagnosis and treatment, which can lead to reduced hospitalization rates and duration (17, 18). Besides, telemedicine has the potential of expanding the medical service to remote areas with poor access to high-quality medical facilities (19). Complementing the provision of healthcare in China, telemedicine is progressively emerging as an alternative of conventional medicine in rural areas, being impactful with limited resources (20). Lastly, during the COVID-19 pandemic, conventional face-to-face care was severely affected, which triggered the urgent need of developing and implementing telemedicine (21).

**Figure 1:**
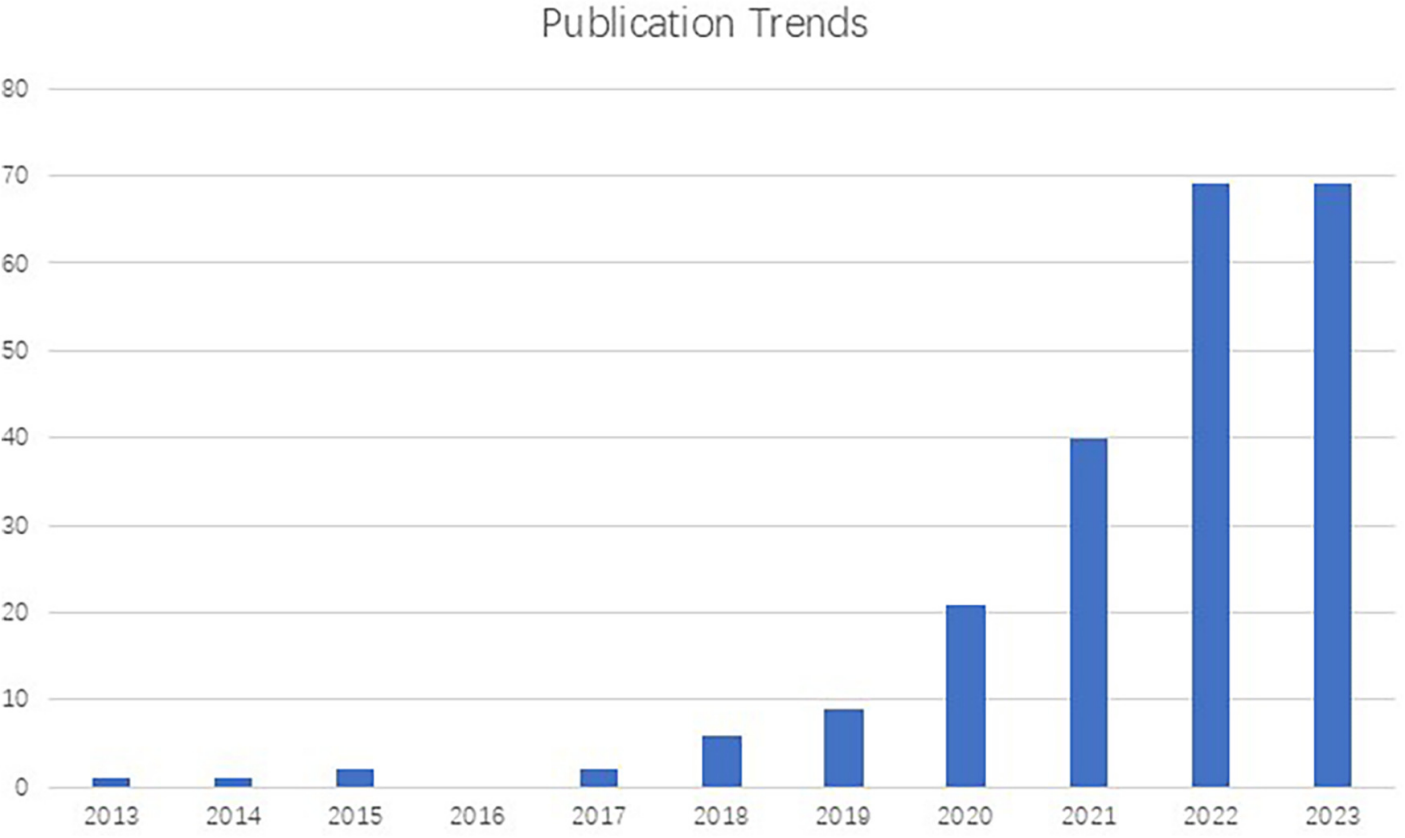
Number of publications on the subject of dermatology and artificial intelligence (2013-2023) registered in PubMed, demonstrating an exponential growth in studies in this field.

Owing to the visual nature of dermatology, dermatological telemedicine has embraced AI-based image identification as a diagnosis support and screening tool (19). In addition, in the pursuit of improving the classification of skin lesions, AI applications have extended to dermatopathology, medical metadata analysis, prognostic prediction, and biomarker screening (22). However, despite the increasing interest in this domain (Figure 1), few articles focus on the advancement of AI integration in dermatology in China, where the prevalence of skin diseases was 26.0% in 2019 (8). In this paper, we provide an overview of Chinese research on the use of AI in dermatology, focusing on machine learning models, datasets, and clinical applications. In addition, the paper addresses the concerns and challenges about the involvement of AI into dermatological clinical practice in China. Furthermore, the relationships between AI and dermatologist are explored, with the insights into the future of the role that AI may play in dermatological practice.

## Methodology

This systematic literature review followed the Preferred Reporting Items for Systematic Reviews and Meta-Analyses (PRISMA) method (23). Databases in English (Pubmed and Web of Science) and databases in Chinese (Wanfang and CNKI) were searched using the keywords (“artificial intelligence” OR “machine learning”) AND (“dermatology” OR “skin lesion”) AND (“image diagnosis” OR “clinical application” OR “segmentation”) AND (“China” OR “Chinese”) for papers from January 2013 to April 2025. This starting point was chosen because the first research in China using AI in dermatology was published in 2013 (24). Exclusion criteria were as follows: (1) conference articles, reviews, or editorials; (2) irrelevant to China; (3) irrelevant to dermatology; (4) without use of AI; (5) no access to full text.

**Figure 2:**
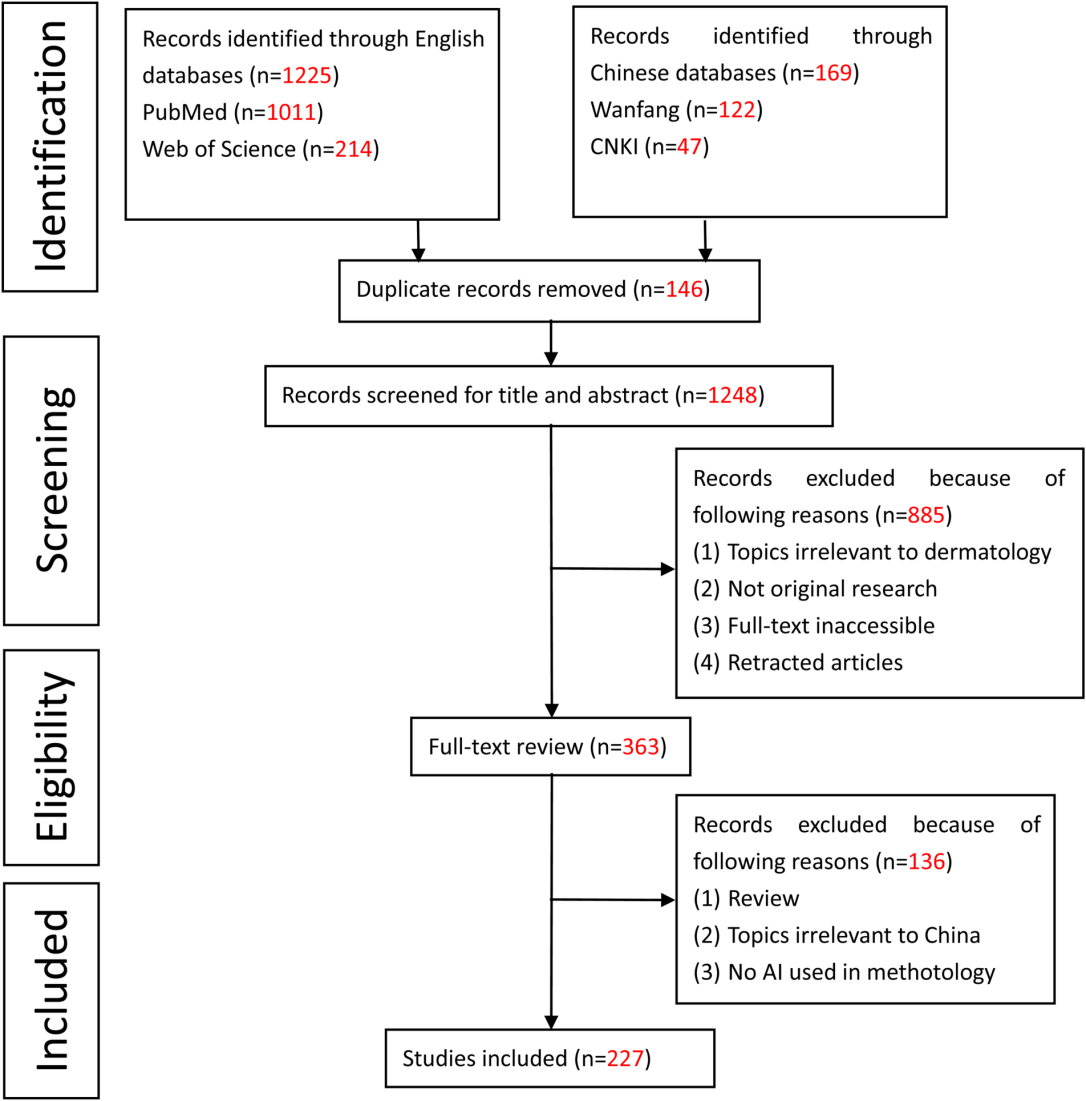
PRISMA graph for the systematic review.

In total, 1394 publications were extracted from these four databases to the EndNote citation manager. 146 duplicates were excluded, leaving 1248 articles. According to the title and abstract screening, 885 articles were removed as non-relevant. Furthermore, 363 articles were reviewed by their full text, of which 136 were excluded because of the following reasons: (1) Review; (2) Topics irrelevant to China; (3) No AI used in the methodology. Finally, a total of 227 articles were selected for the present systematic review (Figure 2). The review of the articles took place by two independent researchers (IOC and YJ) and any contradicting opinions were resolved by a third independent reviewer (ZK and HW). The selected articles were then divided into two groups based on the datasets (public datasets or clinical datasets) used to train the AI model.

## Results

### General study characteristics

In total, 227 articles were reviewed, comprising 25 Chinese and 202 English articles. Among these, 81 focused on single-disease research, while 146 studied multiple skin diseases. Notably, melanoma emerged as the most extensively studied disease in single-disease research (*n* = 46), followed by acne (*n* = 9), psoriasis (*n* = 5), vitiligo (*n* = 3), monkeypox (*n* = 3), actinic keratosis (*n* = 2), and atopic dermatitis (*n* = 2). Only a minority of researches investigating multiple skin diseases studied more than 8 diseases (11/146, 7.5%), with one study analyzing up to 14 diseases. Regarding the objectives of AI models, 132 were designed for classification, 66 for segmentation, and 10 for both segmentation and classification. Additionally, 12 articles aimed at predicting prognosis or treatment effects, 5 focused on screening for biomarkers or medication targets, and 2 were dedicated to image enhancement. According to the dataset used, the studies were categorized into two groups. The group employing public datasets comprised 151 studies (66.5%), while the group employing clinical datasets included 76 studies (33.5%).

### Performance of AI models in skin lesion segmentation

Image segmentation serves as the foundational and pivotal step in medical image analysis (25). Accurate segmentation is vital for both training AI models and ensuring correct classification (26). According to a previous commentary on medical image segmentation, evaluation metrics employed in this sector include the Dice Similarity Coefficient (DSC), Intersection-over-Union (IoU, also known as Jaccard Index), sensitivity, specificity, and accuracy (27). Given the variance in AI performance across different datasets, a quantitative analysis of different AI models should be conducted on the same dataset (28, 29). ISIC 2018 dataset was chosen due to its more uniform sample distribution, making it suitable for both training and testing AI algorithms (30). Consequently, inclusion criteria for evaluating AI models in segmentation were listed as follows: trained and tested on the ISIC 2018 dataset, encompassing all the 5 evaluation metrics above, and addressing all the 7 diseases in ISIC 2018. Out of 66 articles focused on segmentation, a total of 17 studies met these inclusion criteria.

Among the studies included in this sector, CNN emerged as the most frequently utilized model (13/17, 76.5%), followed by Transformer (4/17, 23.5%) and Attention Network (2/17, 11.8%). Inspired by the structure of the animal visual cortex, CNNs are deep learning algorithms well-suited for processing image data, making them widely used in medical imaging fields such as dermatology, radiology, and pathology (48). A notable study introduced a CNN model with a multi-scale design, incorporating a Spatial Adaptation Module to minimize the loss of spatial location information and a Multi-scale Decoding Fusion Module to integrate information across different layers. Overall, this model design prevented the loss of crucial data during segmentation, resulting in more precise segmentation outcomes (43).

Despite the outstanding performance of CNN models in skin lesion segmentation, traditional CNN models often struggle to effectively capture contextual information (49). In light of this, Bahdanau et al. introduced a neural machine translation model with an attention mechanism, enabling the model to concentrate on relevant parts of the input sentence, thereby enhancing translation quality (50). Building on this concept, Attention Networks were subsequently used in biomedical image segmentation to gather contextual information across both long and short distances (51). Dong Y et al. proposed an Attention Network incorporating a combination of feedback fusion blocks and attention mechanism blocks to aggregate feature mapping. The model exhibited improvements compared to state-of-art models without any data augmentation (32).

Transformer, an algorithm that builds on attention mechanisms, was developed to address the limitations of CNN in modeling sequential data (49). Initially applied to Natural Language Processing (NLP) tasks, Transformer has since been utilized in multi-organ segmentation, exhibiting competitive results to state-of-the-art methods (52, 53). It is worth noting that Transformer has a better parameter efficiency, which can be particularly advantageous on the devices with limited computational resources (54). He X et al. proposed a Fully Transformer Network, yielding improved sensitivity and specificity. With its linear computational complexity, the model required fewer computing and memory resources, surpassing CNN models in computational efficiency (35). Additionally, Transformer can also work together with CNN, Chen W et al. introduced a fusion module that combines the strengths of Transformer in extracting global information and those of CNN in extracting local information, resulting in superior segmentation outcomes (40). To overcome the limitations of traditional Transformer and CNNs in challenging lesions, Fan C. et al. applied adaptive spatial-channel attention to reduce Transformer self-attention complexity and preserve spatial-channel interactions, while enhancing local feature extraction via cross-space multiscale attention. Ultimately, their model outperformed CNN/Transformer hybrids in datasets with irregular lesions and noisy backgrounds (47) (Table 1).

**Table 1:** A summary table of the performance of AI models in skin lesion segmentation.

Study	Year	Model	DSC	IoU	SEN	SPE	ACC
Lei B et al. (31)	2020	GAN	0.885	0.824	0.953	0.911	0.933
Dong Y et al. (32)	2021	Attention Network	0.912	0.840	0.899	0.981	0.968
Bai R et al. (33)	2022	CNN	0.915	0.853	0.926	0.980	0.976
Dong Y et al. (34)	2022	CNN and Transformer	0.908	0.836	0.906	0.979	0.967
He X et al. (35)	2022	Transformer	0.898	0.828	**0**.**962**	0.975	0.966
Hu K et al. (36)	2022	Attention Network	0.896	0.831	0.931	0.947	0.957
Jiang Y et al. (37)	2022	CNN	0.934	0.879	**0**.**962**	0.950	0.956
Wang RX et al. (38)	2022	CNN	0.905	0.843	0.911	0.972	0.965
Zhang Z et al. (39)	2022	CNN	0.935	0.882	0.952	0.966	0.959
Chen W et al. (40)	2023	CNN and Transformer	0.908	0.843	0.919	0.973	0.969
Han Q et al. (41)	2023	CNN	0.914	0.859	0.930	0.977	0.963
Jiang Y et al. (42)	2023	CNN	0.935	0.881	0.950	0.968	0.959
Jiang Y et al. (43)	2023	CNN	0.936	0.885	0.950	0.966	0.957
Liu LZ et al. (44)	2023	CNN	0.902	0.832	0.909	0.977	0.944
Yu Z et al. (45)	2023	CNN	0.902	0.836	0.907	0.967	0.967
Zhang W et al. (46)	2023	CNN	0.908	0.835	0.886	0.983	0.961
Fan C. et al. (47)	2025	CNN and Transformer	**0**.**942**	**0**.**892**	0.953	**0**.**990**	**0**.**979**

Bold values indicate statistically significant values.

SEN, sensitivity; SPE, specificity; ACC, accuracy; GAN, Generative Adversarial Network.

### Performance of AI models in multi-disease classification of skin lesions

Previous studies have demonstrated the potential of deep learning algorithms in binary classification of skin lesions, achieving performance comparable to or superior to that of dermatologists (55, 56). However, these methods are not directly translatable to the multi-class classification requirements of clinical settings (57). To extend the application of AI into real-world scenarios, the ability of AI models in multi-disease classification should be evaluated.

HAM10000 dataset encompasses 7 common skin diseases, including Melanoma, Melanocytic nevus (MN), Basal cell carcinoma (BCC), Actinic keratosis (AK), Benign keratosis (BKL), Dermatofibroma (DF), and Vascular lesion (VASC) (58). The Test 3 dataset of ISIC 2018 includes HAM10000, with only minor changes (59). To assess the efficacy of AI models in multi-disease classification, we reviewed and included 13 articles focusing on multi-classification trained and tested on ISIC 2018 or HAM10000. Classification evaluation metrics such as accuracy, sensitivity, specificity, F1-score, and Area Under the ROC Curve (AUC) were employed. Additionally, the learning strategies adopted in these articles were highlighted (Table 2).

**Table 2:** A summary table of the performance of AI models in multi-disease classification of skin lesions.

Study	Year	Model	ACC	SEN	SPE	F1	AUC	TL	EL
Gong A et al. (28)	2020	CNN	0.926	0.484	0.978	0.491	**0**.**918**	Y	Y
Qin Z et al. (60)	2020	CNN	**0**.**952**	0.743	0.966			Y	N
Iqbal I et al. (57)	2021	CNN	0.888	0.888	0.957			N	N
Shan P et al. (61)	2022	CNN	0.893					Y	N
Yao P et al. (29)	2022	CNN	0.875	0.819	**0**.**980**			Y	N
He X et al. (35)	2022	Transformer	0.927	0.857	0.936		0.897	Y	N
Liu Z et al. (62)	2023	CNN	0.873	0.873	**0**.**980**			N	N
Wang G et al. (63)	2023	CNN	0.912	**0**.**951**		**0**.**889**		Y	Y
Wang L et al. (64)	2023	CNN	0.841					Y	N
Yue G et al. (65)	2023	CNN	0.860			0.754	0.872	Y	N
Zhou S et al. (66)	2023	CNN	0.934	0.724	0.925	0.644		Y	N
Hu Z et al. (67)	2024	CNN	0.940	0.917	0.982	0.91	0.993	Y	N
Wan Z et al. (68)	2025	LLM	0.578			0.63		Y	N

Bold values indicate statistically significant values.

ACC, accuracy; SEN, sensitivity; SPE, specificity; F1, F1-score; TL, transfer learning; EL, ensemble learning.

The majority of studies reviewed in this sector adopted transfer learning strategy (11/13, 84.6%). Transfer learning is a machine learning approach that involves reusing an AI model pre-trained for a specific task on a new domain (69). With the advantages such as shorter training time, smaller dataset, and reduced inductive bias, transfer learning is getting increasingly popular in medical fields (70, 71). The application of transfer learning strategy has also extended to AI-CAD of skin lesions. Jain S et al. assessed 6 transfer learning networks on HAM10000 dataset and demonstrated that the Xception Net, pre-trained on the ImageNet dataset, achieved the highest accuracy of 0.905 (72). In China, Qin Z et al. developed a framework using a skin lesion style-based generative adversarial network (GAN) model to generate high quality images, which were then utilized to train another pretrained CNN model (ResNet 50) as the classifier. The image augmentation significantly promoted the performance of CNN model, achieving the highest accuracy of 0.952 among the articles reviewed (60). Instead of using CNN models, He X et al. adopted the Transformer model. Following segmentation, they further applied their Transformer Network to classification. By pre-training their model on the segmentation dataset, they managed to enhance the classification accuracy from 89.6% to 92.7% (35). In light of the promising potential of large language models (LLMs) in medical fields, Wan Z et al. compared the performance of two LLMs (ChatGPT-4 and LLaVA-1.6) in skin disease identification. Although LLMs exhibited lower accuracy compared to state-of-the-art Transformers, they were less biased across different sex and age groups (68).

Ensemble learning, another machine learning approach, involves combining multiple learning algorithms to improve performance (73). With the potential to ensemble the strength of different AI models, the application of ensemble learning has extended to skin lesion diagnosis (74). For instance, Zillur et al. employed this strategy by combining five deep CNN models to classify skin lesions on ISIC 2019 and HAM10000. They proposed a weighted average ensemble learning model, achieving the recall outperforming the other existing systems (75). Among the 11 studies reviewed, 2 utilized ensemble learning methods. Gong A et al. employed the strategy of maximizing individual advantage and block-integrated voting. By integrating binary classification voting of CNN models, the ensemble model outperformed individual CNN in multi-disease classification tasks. Notably, they also used GAN to create a more balanced training dataset (28). In the other study, Wang G et al. combined two network models for feature fusion and incorporated a multi-receptive field module to capture pathological features. The ensemble network achieved classification performance comparable to that of state-of-art models (63). However, it is important to note that ensemble learning demands significant computing resources and can result in long response time, making it challenging to implement on portable devices (76). To address this issue, Yan P et al. designed a model combining a new loss function and cumulative learning strategy. This non-ensemble model demonstrated performance comparable to that of ensemble models with lower computing burden and less computing time (29).

### Public datasets used in AI training

The preparation of dataset stands as the primary and fundamental step of AI training (77). The quality of the dataset is important to the establishment of AI models, especially to segmentation and classification tasks (78). The appropriate selection of dataset can greatly enhance the ability and generalizability of AI models (79). Among the 144 studies trained on public datasets, ISIC 2018 is the most commonly used dataset (58/144, 40.3%), followed by ISIC 2017 (57/144, 39.6%), ISIC 2016 (35/144, 26.4%), PH2 (33/144, 22.9%), ISIC 2019 (13/144, 39.6%), and HAM 10000 (12/144, 8.3%). Some of the articles used more than one dataset. The ISIC datasets, provided by the International Skin Imaging Collaboration (ISIC), are widely used resources in dermatology imaging research (80). These datasets were established for the public benchmark challenges on dermoscopic image analysis held annually from 2016 to 2020 (81). The HAM10000 dataset, also known as “Human Against Machine with 10,000 training images,” comprises a comprehensive collection of dermoscopic images, which are included in ISIC 2018 (82). The PH2 dataset, named after the Hospital Pedro Hispano (PH) where it was compiled, consists of dermoscopic primarily used in research related to CAD of melanoma (83) (Table 3). More features of the most frequently used public datasets are listed below:

**Table 3:** Table of public datasets used in AI training.

Dataset	Year	Data Size	Reference
PH2	2013	40 Melanoma, 160 Nevus	(83)
ISIC 2016	2016	248 Malignant; 1029 Benign	(84)
ISIC 2017	2017	521 Melanoma; 386 seborrheic keratosis; 1,843 benign nevi	(80)
ISIC 2018	2018	2594 for segmentation, 10003 for classification (1110 Melanoma, 6698 MN, 514 BCC, 130 AK, 1097 BKL, 115 DF, 142 VASC, and 197 SCC)	(85)
HAM 10000	2018	1113 Melanoma, 6705 MN, 514 BCC, 327 AK, 1099 BKL, 115 DF, and 142 VASC	(82)
ISIC 2019	2019	4522 Melanoma, 12875 MN, 3323 BCC, 867 AK, 2624 BKL, 239 DF, 253 VASC, 628 Squamous cell carcinoma (SCC)	(86)

The feature extraction ability of CNNs builds on large and balanced dataset. However, the challenge of insufficiency and uneven distribution poses a significant obstacle in AI training (28). The size of these public datasets varies greatly, form 200 images for PH2 to 33,569 images for ISIC 2019. Generally, AI models trained in small dataset are more easily challenged by radical bias (56, 87). Furthermore, the distribution of each skin disease within the dataset can impact the performance of AI models. Class imbalance occurs when one class is much more abundant than the other classes, leading to biased model training, misleading performance metrics, and reduced generalization (88). As shown in the table above, all the public datasets encounter the challenge of class imbalance, which may lead to diminished effectiveness (81). To address this issue, Li Z et al. used data augmentation to expand the images of minority classes and weighted random sampling method to avoid oversampling (89). Qian S et al. adopted an adaptive loss-weighted cross-entropy of specific categories to address significant imbalances across different categories of skin lesion images (90). Additionally, Qin Z adopted a skin lesion style-based GAN to augment the data, resulting in an improvement of accuracy from 0.944 to 0.952 (60).

The quality of dataset plays a crucial role in training AI models for skin lesion segmentation and classification (91). Inaccurate data can lead to impaired model performance and poor reproducibility (92). Biases present in datasets finally result in bias in AI models (93). Within public datasets, ground truth data are provided to identify the edges of skin lesions. However, in ISIC 2016, only binary classification of malignant or benign is provided, which offers limited practical value since not all malignant diseases are melanoma (81). Additionally, it has been reported that a number of images in ISIC 2017 were given incorrect ground truth information (94). The presence of pathologic verification in datasets significantly impacts the reliability of AI classification (95). However, not all of the images in public datasets have corresponding pathologic diagnosis. The rate of pathologic verification of ISIC 2017 was only 26.3%, compared to 53.3% of HAM 10000 (82).

Thus, the generalization of AI models remains a significant concern in the application of AI in medical fields (96). Most of the studies reviewed in this article tested their models on the same dataset where they were trained, which may not reflect the performance in real-world contexts. Models trained in one dataset could show lower performance when tested on another dataset because of the differences in image settings (2). The performance of AI models relies on these details, and even minor deviations have the potential to cause considerable bias (97). Therefore, data pre-processing and image normalization are crucial to reduce the interference of deviations in datasets (28). For example, Tao S et al. converted the dermoscopic images provided by ISIC 2017 dataset from RGB format to HSV format, which is less influenced by the changes in external lighting. As a result, their model trained on ISIC 2017 exhibited great trans-dataset effectiveness when tested on PH2 (98). Despite the progress made by researchers, the application of these AI-CAD tools is still under question, and their performance should be evaluated extensively in real-world scenarios.

### Performance of AI-aided skin lesion classification on clinical datasets

It is worth noting that none of these public datasets mentioned above collect data from Asian countries (99). Previous researches have demonstrated that AI models may not achieve favorable results when transferred to a dataset collected from a population on which the AI models were not trained (100). Consequently, AI models trained on public AI datasets may not adequately address clinical needs in China. In addition to public datasets, many Chinese researchers opt to train and test their models on the dataset developed in local hospitals, collected via dermoscopy, camera, or smartphone. The comparison between AI and clinical physicians is widely discussed and has garnered intensive attention. In 2019, a comparative study was performed between 139 state-of-art machine learning algorithms and 511 dermatologists worldwide, demonstrating that the performance of AI classifiers surpassed that of experts with more than 10 years of experience (101). In this section, we include the articles that (1) were performed on self-developed clinical datasets; (2) focused on skin lesion classification; and those that (3) included a comparison between AI models and dermatologists. Further details are listed in Table 4 below.

**Table 4:** Summary table of the performance of AI-aided skin lesion classification on clinical datasets.

Study	Year	Disease	Algorithm	Type of image	Performance of AI	Performance of dermatologists
Chang W et al. (24)	2013	Melanoma	SVM	Conventional Photographs	ACC = 0.906 SEN = 0.856, SPE = 0.876	ACC = 0.833, SEN = 0.859, SPE = 0.853 (NS)
Xie B et al. (102)	2019	BCC and MN	CNN	Conventional Photographs	ACC = 0.92	ACC = 0.895 (NS)
Huang K et al. (103)	2020	BCC and Seborrheic Keratosis	CNN	Conventional Photographs	ACC = 0.856 SEN = 0.857 SPE = 0.857	TPR = 0.845, FPR = 0.114 (SP)
Li C et al. (104)	2020	11 Skin Diseases	CNN	Dermoscopic and Conventional Photographs	ACC = 0.764	ACC = 0.634 (NS)
Wang S et al. (105)	2020	4 Skin Diseases	CNN	Dermoscopic Image	SEN = 1.000, SPE = 0.605	SEN = 0.872, SPE = 0.838 (NS)
Yang Y et al. (106)	2021	6 PSLs	CNN	Conventional Photographs	SEN = 0.932, SPE = 0.989	SEN = 0.908, SPE = 0.982 (NS)
Yang Y et al. (107)	2021	Psoriasis and other papulosquamous skin diseases	CNN	Dermoscopic Image	SEN = 0.869, SPE = 0.956	SEN = 0.732, SPE = 0.912 (SP)
Zhang L et al. (2)	2021	Vitiligo	CNN	Conventional Photographs	F1 = 0.968, SEN = 0.972, SPE = 0.962	F1 = 0.893, SEN = 0.811, SPE = 0.999 (SP)
Zhao Z et al. (3)	2021	Rosacea and Acne	CNN	Conventional Photographs	ACC = 0.890, PRE = 0.867	ACC = 0.913, PRE = 0.881 (SP)
Zhu C et al. (108)	2021	14 Skin Diseases	CNN	Conventional Photographs	ACC = 0.928, SEN = 0.835, SPE = 0.941	ACC = 0.921, SEN = 0.685, SPE = 0.955 (NS)
Guo L et al. (109)	2021	Vitiligo	CNN	Conventional Photographs	ACC = 88.66%, SEN = 88.00%, SPE = 89.36%	ACC = 92.78%, SEN = 97.33%, SPE = 87.94% (NS)
Ding H et al. (110)	2022	6 PSLs	CNN	Conventional Photographs	PRE = 0.956, SEN = 0.962, SPE = 0.952	PRE = 0.994, SEN = 0.987, SPE = 0.981 (NS)
Ge L et al. (111)	2022	Acne, Rosacea, and Dermatitis	GBM	Dermoscopic Image	ACC = 84.4%	ACC = 35.5% (RD)
Yu Z et al. (112)	2022	Scalp Psoriasis and Seborrheic Dermatitis	CNN	Dermoscopic Image	SEN = 0.961, SPE = 0.882	SEN = 0.745, SPE = 0.882 (SP)
Zhu X et al. (113)	2022	7 Nail Diseases	CNN	Dermoscopic Image	SEN = 0.930, SPE = 0.785	SEN = 0.678, SPE = 0.766 (SP)

ACC, accuracy; SEN, sensitivity; SPE, specificity; F1, F1-score; PRE, precision; TPR, ture positive rate; PR, faux positive rate; NS, not specified; SP, senior physician; RD, resident doctor; SVM, support vector machines; GBM, gradient boosting machine.

Among the 15 studies included, 13 articles used a CNN model, while one article employed Support Vector Machines (SVM) and another used Gradient Boosting Machine (GBM). The skin diseases included in these studies ranged from single-disease classifications such as Melanoma to multiple-disease classifications of up to 14 diseases. 5 studies trained their model only with dermatologic images, while 9 studies trained their models with conventional images. Additionally, one study trained their model with both dermatologic images and conventional images. 6 studies claimed that their models achieved results comparable to those of experienced senior dermatologists. 8 studies claimed that the performance of their models was comparable to those of dermatologists, although the raters were not detailed. One study claimed their models achieved results significantly superior to those of resident doctors.

In 2013, Chang W et al. conducted the first AI-CAD research in China, employing SVM, a machine learning method used for classification and regression tasks, to eliminate the least informative features. The model achieved an accuracy comparable to that of clinicians. However, it was still limited to binary classification of benign or malignant lesions (24). Huang K et al. utilized a large-scale Xiangya Derm dataset established by Xiangya Hospital, consisting of 107,565 clinical images encompassing 541 types of skin diseases (114). Focusing on the binary classification of BCC and SK, they compared the performance of InceptionResNetV2 with that of dermatologists, demonstrating that the model was comparable to the average of 13 general dermatologists (103). Zhu C. et al. conducted a study using a self-developed dataset consisting of 13,603 dermoscopic images covering 14 diseases. They employed a fine-tuned EfficientNet-b4 model and received comparable results compared to 280 dermatologists of all levels on the classification task of 8 diseases (19).

In addition to comparing AI with dermatologists, some researchers are exploring deep learning models as assistants to dermatologists. Shen Y et al. developed a deep learning model to classify 22 common skin diseases, achieving a top-1 accuracy of 45.05%. They further developed an online dermatology diagnosis application based on the JAMA CLEAR dermatology guidelines. The AI diagnostic tool achieved a 63.04% acceptance rate among physicians across 18 tertiary hospitals (115).

In recent years, the application of Vision-Language Models (VLMs) has attracted growing attention in the field of medical diagnosis. The advantage of VLMs lies in their ability to integrate multimodal information from complex medical image data, making diagnoses more accurate and comprehensive (116). To explore the use of VLMs in dermatological diagnosis, Zhou J et al. developed SkinGPT by combining a pretrained vision transformer with an LLM through learning from over 50,000 skin disease images and clinical experiences of dermatologists. When working alongside dermatologists, 80.63% of the diagnoses of SkinGPT were considered useful, indicating that VLM can alleviate the burden on dermatologists by offering rapid suggestions (117).

### Use of AI in dermatology other than skin lesion diagnosis

Due to the shortage of experienced pathologists in fundamental hospitals in China, the pathological diagnosis of rare skin diseases can be challenging (118). AI-based diagnosis has emerged as a potential solution to this issue (119). Zheng T et al. established a deep reinforcement learning model to detect melanoma cells from whole-slide histopathology images (WSI). Due to the lack of available annotations in WSI datasets, the researchers adopted weakly supervised learning, which does not require detailed annotation. The model finally achieved an accuracy, sensitivity and specificity of 0.966, 0.991 and 0.984, respectively (120). Wu H et al. applied pre-trained CNN models to the diagnosis of Extramammary Paget's disease (EMPD) using pathological images from a self-developed dataset. The model achieved an accuracy, sensitivity and specificity of 0.950, 0.923 and 0.9792, respectively (121). Jiang S et al. extended the AI- assisted pathological diagnosis to the classification of 11 types of skin diseases and achieved an accuracy of 86.8%, outperforming existing CNN models (122).

AI can be employed in predicting the prognosis of skin disease. Xue Y et al. developed a prediction model to estimate the probability of anti-melanoma differentiation associated gene 5 (MDA5) antibody, which is a biomarker associated with unfavorable outcomes in juvenile dermatomyositis (JDM) patients. The model was established using Stepwise logistic regression, least absolute shrinkage and selection operator (LASSO) regression, and random forest (RF) method based on clinical records and auxiliary examinations, achieving an AUC of 0.975 (123). Li W et al. established a SVM model to evaluate the risk types of melanoma using mRNA, miRNA, and DNA methylation data, indicating promising ability to identify different risk subgroups (124). Additionally, AI can be used in predicting the recurrence of skin disease. Cao C et al. introduced an RF model to estimate the recurrence rate of dermatofibrosarcoma protuberans (DFSP) based on MRI images. The model achieved the best concordance index score of 0.875, which is superior to Ki67 index (an independent predictor of recurrence) (125).

Finally, AI can serve as a valuable tool in screening for biomarker of skin diseases or new therapeutic targets. For instance, Liu J et al. developed a machine learning model using cuproptosis-related genes to elucidate their role in the metastasis of melanoma. This model successfully predicted the overall rates of melanoma patients and identified seven key genes, along with 98 potential drugs (126). Song J et al. screened ten pyroptosis-related genes using RF model, revealing the significance of pyroptosis in psoriasis and suggesting potential therapeutic targets. This model demonstrated promising results in external validation, with an AUC of 0.852, and unveiled the involvement of metabolic enhancement and the MAPK signaling pathway (127). Similarly, Xing L et al. introduced RF and LASSO methods to screen for psoriasis biomarkers among 33 differentially expressed methylated genes, identifying GJB2 as the potential target gene for the treatment of psoriasis (128).

### AI application in the market

In recent years, an increasing number of AI-aided medical applications have emerged in the market, particularly in China, and some have successfully made their way into clinical settings (129). Many of these applications are accessible via smartphones, facilitating widespread adoption of AI diagnosis (22). The five most used, AI-aided skin lesion diagnosis tools in China are shown on the Table 5 below.

**Table 5:** Summary table of AI-aided medical applications in the market, in China.

Name	Year	Disease	Accessibility	Highlight	Reference
AIDERMA	2018	85 skin diseases	Only to doctors	The first comprehensive skin disease diagnosis platform in China, with an accuracy of 86% for 85 skin diseases and 95% for 34 common skin diseases	(129)
Youzhi AI	2018	Multiple skin diseases	Only to doctors	Based on one of the biggest skin lesion datasets in China, with an accuracy of 0.912 in binary classification of benign and malignant skin tumors and 0.814 in multi-classification of skin diseases	(104)
AIDDA	2020	Psoriasis, Eczema, and Atopic Dermatitis	Only to doctors	Trained on 4,740 clinical images, with an overall accuracy, sensitivity and specificity of 0.958, 0.944 and 0.972, respectively	(130).
VoxelCloud DermX	2020	143 skin diseases	Open to public	The top1, top3, and top5 diagnostic accuracies of the models are 71%, 89%, and 94%, respectively	(131)
SkinTeller	2023	Psoriasis	Only to doctors	Designed to evaluate the Psoriasis Area and Severity Index (PASI), achieving results better than the average of dermatologists	(132)

AIDERMA, Youzhi AI, and VoxelCloud DermX are capable of detecting multiple skin lesions, covering most of common skin diseases. Among them, VoxelCloud DermX stands out for having the broadest range of skin disease classifications. Most the diagnosis tools above are accessible only to clinical doctors, which does not meet the demand of self-diagnosis by patients. VoxelCloud DermX is open to public and can serve as a screening tool for skin malignant cancer. However, no large-scale research has been performed to evaluate the performance of VoxelCloud DermX in real world scenarios. Following diagnosis, both AIDERMA and VoxelCloud DermX provide continuing education, treatment guidance, and auxiliary consultation (129). Notably, none of these AI applications have been widely adopted in clinical frontlines, indicating that the use of AI-CAD tools is still in its early stages in China. Further evaluation of reliability is necessary through large-scale clinical trials.

## Discussion

### Challenges of integration of AI into clinical settings

With the increasing interest in telemedicine following the COVID-19 pandemic, self-diagnosis on AI diagnosis platforms has garnered significant attention when conventional medical care was out of reach (133). However, it's worth noting that most of the frequently used pubic datasets and some of the clinical datasets consist only of dermoscopic images collected and processed exclusively by dermatologists, which may not reflect real-world scenarios. Dermoscopy, a widely used technique in dermatology, provides dermatologists with a detailed view of skin lesions in high resolution, significantly improving diagnostic accuracy (134). AI models also tend to perform better when trained on dermoscopic images compared to conventional photographs (104). However, for the dermatologists in restricted-resources areas and general practitioners who have limited access to dermoscopy, using AI-CAD with dermoscopy may not be realistic. If AI tools are only trained and tested with dermoscopic images, their effectiveness on conventional datasets may be questionable. Meanwhile, many clinical datasets use cameras, including smartphones, to capture clinical pictures (135, 136). For example, YOLOv5 model has shown good performance on untrained datasets consisting of images taken by smartphone (110). These practices highlight the potential of AI in smart device-based self-diagnosis.

Due to the opacity of the process, deep learning models have long been regarded as a black box, casting doubt on its integration into real-world applications (137). To address this issue, future AI models should be more transparent and explainable. To improve the interpretability, Wang S et al. introduced the interpretability modules into their multimodal CNN model, providing diagnosis along with the visual analysis for explanations (138). Huang K et al. performed the evaluation methods to explore the interpretability of their model. They compared the Gradient-weighted Class Activation Mapping (Grad-CAM) and Local Interpretable Model-Agnostic Explanations (LIME), which are able to highlight the regions of image that lead to the decision of CNN models. Their findings demonstrated that their model was interpretable, with LIME showing more accurate results in localizing the responsible region in AI diagnosis compared to Grad-CAM (103).

Machine learning algorithms are built on data. Therefore, any biases present in the training dataset can result into biases in the model (139). These biases may stem from underrepresentation of certain patient subgroups within the dataset (140). For example, most of the AI skin lesion diagnosis models have been trained on datasets composed of individuals with light skin tones. Consequently, these models tend to perform significantly worse when tested by the skin lesion images from individuals with dark skin compared to those with light skin (119). However, the sharing of data across centers raises concerns regarding data privacy, as these datasets may contain sensitive patient information. One possible solution to this dilemma is federated learning, a machine learning strategy that allows AI models to be trained across different datasets without exchanging the data itself (13). So far, federated learning has shown promising results in various fields such as radiology and oncology, offering a potential option to address bias while protecting patient privacy (141).

### Methods to improve the performance of AI in clinical application

The quality of images greatly influences the training and performance of AI models (142). Datasets often contain images from diverse sources, indicating the necessity of image pre-processing. Image pre-processing involves cropping, rescaling, contrast enhancement, noise reduction, and removal of artifacts. Cropping refers to the process of selecting a square region with the skin lesion in the center to minimize possible interference (143). To enhance the training of CNN models, manual cropping of clinical images was performed by Yang Y et al., resulting in a model with comparable sensitivity and specificity to expert dermatologists (106). Given the variation in image resolutions due to diverse data sources, Iqbal I et al. rescaled the images to 64*64 pixels with inter nearest interpolation, which also alleviates the computational burden (57). Contrast stretch is employed to enhance contrast between diseased and normal skin regions (144). Due to the electronic noise brought by the digital camara, the interference of environmental factors, and the movement of patients, noise removal is necessary to image pre-processing. Hu L et al. adopted the Wang-Mendel algorithm, a fuzzy logic-based technique, to generate denoised images (145). Moreover, human hair may appear as an interference in skin lesion pictures (146). To remove the hair, Z. Al-Huda et al. used morphology top-hat to create the hair mask, followed by inpainting TELEA algorithm to fill in the masked area (144).

In clinical settings, dermatologists make the diagnosis based on not only the visual characteristics of skin lesions but also medical information such as age, gender, symptoms, and body site (103). However, public datasets often lack or have insufficient medical data, limiting the generalization and interpretability of AI models. To address this gap, metadata-rich clinical datasets should be developed (99). Cai G et al. proposed a model incorporating two encoders: a Transformer model to extract visual features and a Soft Label Encoder to embed medical data. By fusing these features using a Mutual Attention block, they demonstrated that adding metadata improved accuracy from 0.75 to 0.816 on ISIC 2018 compared to models using only images (135). Ou C et al. introduced a CNN model to extract visual features from smartphone images and a multi-layer perceptron model to extract metadata features. They found that including metadata significantly enhanced model performance, increasing accuracy from 0.616 to 0.768 (147). Similarly, Chen Q et al. developed a model combining clinical images and medical data using feature fusion and attention mechanisms. Integration of metadata improved accuracy from 0.716 to 0.804, highlighting the effectiveness of multimodal data fusion strategies in improving AI model performance (136).

Medical data are often high-dimensional and complex, making comprehensive feature extraction from clinical information challenging. The introduction of VLM into the medical field has enabled the integration of textual and visual data (148). For rare disease cases with limited labeled images, VLMs can be trained using few-shot or zero-shot learning approaches (116). In addition, VLMs generate human-readable reasoning for AI-generated diagnoses, making them suitable as health advisors for providing preliminary diagnoses (117). Although our review indicates that the application of VLMs in Chinese dermatology is still in its early stages, VLMs hold great potential as interpretable diagnostic tools in the medical imaging domain.

### AI assisted diagnosis

While there are concerns about AI potentially replacing human dermatologists, our research indicates that AI still cannot entirely replace them due to various reasons such as ethical concerns, limited disease spectrum coverage, technical limitations, insufficient interpretability, and unaddressed biases. The impressive performance of AI in experimental settings using specific high-quality datasets may not accurately reflect its real-world ability (79). However, AI can still serve as an assistant diagnosis tool to alleviate the burden of dermatologists or enhance the diagnostic accuracy of general practitioners who are not specialized in dermatology.

In general, the dermatologists regard the AI more as an assistant tool rather than a potential rival. According to an international survey conducted in 2019, the majority of the dermatologists (77.3%) agreed that AI would bring innovation to dermatology, while only a minority (5.5%) believed human dermatologist would be replaced by AI (149). Another survey including 1,228 dermatologists across China demonstrated that most of the participants (95.36%) believed that AI should serve as an assistant in diagnosis and treatment. Furthermore, a significant proportion (64.17%) of participants thought that AI should first be implemented in secondary hospitals in China (150).

Several studies have already demonstrated that AI could assist clinical doctors to make better decisions in the diagnosis of skin lesions. Instead of the competition between AI and human, Ba W et al. focused on the potential collaboration of dermatologists and CNN models in skin lesion diagnosis. They found that AI-assisted dermatologists achieved a higher accuracy in the classification of multiple skin diseases, with an improvement of accuracy from 0.628 to 0.766. The dermatologists with less than 10 years of experience benefited more from the assistance of AI (151). Another study using CNN models to improve the performance of three unexperienced doctors in the classification of psoriasis and seborrheic dermatitis, achieving an improvement of AUC from an average of 0.571 to 0.805 (112).

### Limitations

Meta-analysis couldn't be conducted due to diverse methodologies across studies and absence of specific evaluation metrics. As we wanted to focus on the application of AI in clinical setting, not all of the algorithms were introduced in detail. We extracted data from Web of Science, pubmed, CNKI, and Wanfang, but no gray literature was included. Clinical trials were not included as no AI-based skin lesion diagnosis tools were known to have undergone clinical trials in China. Certain skin conditions such as burn wounds and diabetic foot ulcers were not included because they were related to clinics and specialties other than dermatology. As this study concentrated on AI use in Chinese dermatology, some articles of international collaborations were excluded if neither the first author nor the corresponding author was Chinese and conducting work in China. The majority of studies reviewed trained their model on public dataset. However, as most of the public datasets are not China-centric, these studies may not specifically reflect the application of AI in China. To address this discrepancy, we also included and evaluated diagnostic tools trained in Chinese clinical datasets, but these datasets often lack clarity and accessibility.

## Conclusion and prospective

Our study offers an overview of the ongoing progress in AI within the field of dermatology in China. While AI has demonstrated comparable performance to human experts in diagnosing multiple skin diseases, its clinical implementation remains at an early stage due to limitations in generalizability and interpretability. Large-scale clinical trials are warranted to further validate its efficacy in real world. However, the potential of AI in dermatology is promising. Given China's shortage of medical resources and the significant clinical burdens faced by physicians, AI technology is viewed as a solution to address healthcare disparities in remote areas. As a useful assistant to physicians, AI has the potential to enhance the quality of medical care, reduce costs, and alleviate the burden of dermatologists.

## Data Availability

The original contributions presented in the study are included in the article/Supplementary Material, further inquiries can be directed to the corresponding author.
